# Correspondence

**Published:** 1918-06

**Authors:** 


### CORRESPONDENCE

**This department is intended for the presentation of news and views not coming within the scope of other departments, and particularly to afford opportunity for discussion and criticism of views elsewhere expressed.**

To the Editor of the Buffalo Medical Journal:

Careful investigation of the status of a Christian Science of Grace M. Trankla, notice of whose suit against Clarence C. Burger to recover pay for services as a practitioner was published in your March number, discloses that she is not known as a member or regular attendant of the local Churches, that she is not a member of The Mother Church in Boston, nor on the list of authorized practitioners. The methods indicated in the complaint are not in accordance with the custom and practice of the true followers of this teaching; in short they are positive proof that she is not a Christian Scientist.

In regard to remuneration for services, authorized practitioners on Christian Science are guided by the By-law in the Manual on The Mother Church, by Mary Baker Eddy, which says, "A member of The Mother Church shall not, under pardonable circumstances, sue his patient for recovery of payment for said member's practice, on penalty of discipline and liability to have his name removed from membership. . . A Christian Scientist is a humanitarian; he is benevolent, forgiving, long-suffering, and seeks to overcome evil with good." (Article VIII, Section 22.)

Very truly yours,
ALBERT R. GILMORE,
Committee on Publication.

---

1. Attention of medical officers is directed to the provisions of paragraph 423, M. M. D.—"Medical Officers will not publish professional papers requiring reference to official records or to experience gained in the discharge of their duties without the previous authority of the Surgeon General."
2. Numerous scientific papers written by officers of the medical department have recently appeared in the medical press without specific authority from this office. This practice will be discontinued, and the above regulation will be strictly complied with.
3. Officers desiring publication of professional papers will submit two copies to the Surgeon General with request for permission to publish same. Upon approval, a copy will be forwarded to the journal designated by the officer for publication.

By direction of the Surgeon General:

C. L. FURBUSH,
Lieutenant Colonel,
Medical Corps, N. A.

